# Unveiling the Microeukaryotic Landscape of the Red Coral *Corallium rubrum* Across the Northwestern Mediterranean Sea

**DOI:** 10.1111/1758-2229.70227

**Published:** 2025-11-25

**Authors:** Camille Prioux, Doria Filipponi, Christine Ferrier‐Pagès, Denis Allemand, Romie Tignat‐Perrier

**Affiliations:** ^1^ Centre Scientifique de Monaco Monaco Principality of Monaco; ^2^ Unité de Recherche sur la Biologie des Coraux Précieux CSM—CHANEL, Centre Scientifique de Monaco Monaco Principality of Monaco; ^3^ Sorbonne Université, Collège Doctoral Paris France

**Keywords:** eukaryome, geographical patterns, marine animal forests, Mediterranean octocorals, metabarcoding, microbiome, temperate corals

## Abstract

Octocorals such as *Corallium rubrum* are key components of temperate Marine Animal Forests (MAFs), providing three‐dimensional habitats that support diverse marine life. However, *C. rubrum* faces growing threats from overexploitation and climate stressors such as ocean warming. While the coral's bacterial microbiome is well‐documented and stable across spatial and temporal scales, the associated microeukaryotes, collectively referred to as the coral eukaryome, remain poorly characterised. In this study, we used *18S rRNA* gene metabarcoding to explore the eukaryome of 46 *C. rubrum* colonies collected from five sites (~44,670 km^2^) in the northwestern Mediterranean. We identified a limited set of core microeukaryotic families, including Licnophoridae and Dino‐Group I Clade 1, which were present at all sampling locations. Despite sharing core taxa, eukaryome composition showed high variability between sampling sites, seasons and years. This suggests the red coral eukaryome is strongly influenced by local environmental factors. Given the increasing frequency of marine heatwaves and habitat degradation, further research is needed to understand the ecological roles of key eukaryotic taxa and their contribution to coral holobiont resilience. Clarifying the function of the eukaryome is essential for predicting how *C. rubrum* and other habitat‐forming octocorals will respond to future climate scenarios.

## Graphical abstract

This study demonstrates that the eukaryome of the Mediterranean octocoral *Corallium rubrum* exhibits high variability, and the dominance of certain taxa is influenced by spatiotemporal environmental factors. Despite this, core microeukaryotic families, such as Licnophoridae and Dino‐Group I Clade 1, were consistently present, suggesting key roles in the coral's holobiont.

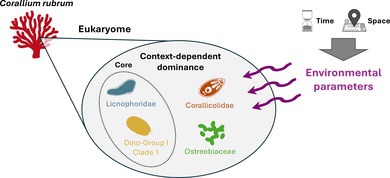

## 1 Introduction

Marine Animal Forests (MAFs) are unique habitats and biodiversity hotspots (Casas‐Güell et al. 2015; Roberts et al. 2002; Ponti et al. 2019), offering shelter to diverse marine species (Piazzi et al. 2021) and providing valuable services such as fisheries support, coastal protection, and tourism (Paoli et al. 2017; Worm et al. 2006; Rossi et al. 2017). Among these, the shallow and upper‐mesophotic Marine Animal Forests (depths < 60 m) of the Mediterranean Sea stand out as ecologically important ecosystems, primarily dominated by sponges and habitat‐forming octocorals such as *Eunicella* spp., *Paramuricea clavata* , and the iconic red coral *Corallium rubrum* (Ballesteros 2006; Ponti et al. 2019; Gori et al. 2011). Like all meta‐organisms, corals form complex holobionts with diverse microorganisms that collectively make up the microbiome, an essential component for maintaining health through nutrient cycling, resistance to stress and protection from pathogens (Voolstra et al. 2024; van de Water, Allemand, et al. 2018). In the literature, the aforementioned functions are usually associated with the photosynthetic dinoflagellates from the family Symbiodiniaceae and the associated bacterial communities, which are the most studied symbionts of the coral microbiome. Still, the complex coral‐associated microbial communities contain other microeukaryotic members (referred to as the “eukaryome”) that have been poorly investigated despite their likely relevance for the coral holobiont (del Campo et al. 2020; Bonacolta et al. 2023). This is mainly due to the large difficulty of culturing microeukaryotes, as well as the technical limitations of using *18S rRNA* gene‐based sequencing to study coral‐associated microeukaryotes (del Campo et al. 2019). Because both the microeukaryotes and their coral hosts are eukaryotes, the metabarcoding approach often results in data that is dominated by host DNA. To minimise this issue, specific methodological strategies have been developed, including optimised primer design and the use of host‐specific blocking oligonucleotides (del Campo et al. 2019; Parfrey et al. 2014; Wilcox et al. 2014).

Recent DNA‐based studies have emphasised the importance of coral‐associated microeukaryotes in terms of species richness and abundance. Some microeukaryotes were frequently reported to be associated with corals and are suggested to be important players of the coral microbiome. For example, Corallicolidae apicomplexans were found in high prevalence across all major groups of corals (Kwong et al. 2019, 2021; Bonacolta et al. 2023, 2024). In Kwong et al. (2019), the apicomplexan *ARL‐V 16S rRNA* gene was recovered in 83% of the 43 coral samples, covering 38 coral species. However, to date, only a few studies have examined the diversity of the eukaryome in Mediterranean octocorals (Bonacolta et al. 2024; Prioux, Ferrier‐Pagès, Del Campo, et al. 2025; Prioux, Ferrier‐Pagès, Lamarca, et al. 2025), and these have focused exclusively on conditions of thermal stress. As a result, our understanding of octocoral‐microeukaryotes associations under baseline, non‐stressed conditions remains limited. They identified a large richness of microeukaryotes with up to 8187 ASVs (Amplicon Sequence Variant) and 3181 ASVs in association with colonies of *C. rubrum* and different populations of *P. clavata* , respectively (Prioux, Ferrier‐Pagès, Del Campo, et al. 2025; Bonacolta et al. 2024). Some dominant microeukaryotes were found in the samples of both species such as the Syndiniales Dino‐Group I, the Licnophoridae ciliates and the Corallicolidae apicomplexans (Prioux, Ferrier‐Pagès, Del Campo, et al. 2025; Bonacolta et al. 2024). While these studies are pioneering and provide new insights on the large diversity of microeukaryotes associated with Mediterranean octocorals, further studies are necessary to get a better understanding of the dynamics of the octocoral‐microeukaryotes association and identify key microeukaryotic members.

Previous studies have shown that *C. rubrum* maintains stable associations with specific bacterial symbionts (i.e., Spirochaetaceae bacteria) across both temporal and geographical scales (van de Water et al. 2016, 2018). This knowledge has enabled the following investigations to focus on these specific symbionts (Tignat‐Perrier et al. 2023, 2025; Prioux et al. 2023, 2024), as they likely play a crucial role in the holobiont functioning. Although the stability and functional significance of bacterial symbionts in corals are well documented, it is still unclear to what extent similar patterns hold true for the eukaryome. It is therefore essential to investigate whether these microeukaryotic associations exhibit stability and functional relevance, in order to fully comprehend their role in coral health, resilience, and the dynamics of the holobiont.

In this study, we analysed samples of *C. rubrum* colonies collected at five different locations (*Porticcio*, *Cap de Creus*, *Cassis*, *Villefranche‐sur‐Mer*, *Portofino*) in the Northwestern Mediterranean Sea covering a total surface of 44,670 km^2^. Here, we investigated the composition of the red coral eukaryome in different red coral populations using an *18S rRNA* gene metabarcoding approach with specific primers designed in Prioux, Ferrier‐Pagès, Del Campo, et al. (2025). These primers exclude the red coral *18S rRNA* sequences, while covering a wide diversity of microeukaryotic taxa. We aimed to gain a better understanding of microeukaryotic diversity associated with the red coral, uncover the stability of the association between *C. rubrum* and its associated microeukaryotes, and assess how local environmental conditions may influence the composition of the red coral eukaryome.

## 2 Methods

### 2.1 Sample Collection

**FIGURE 1:**
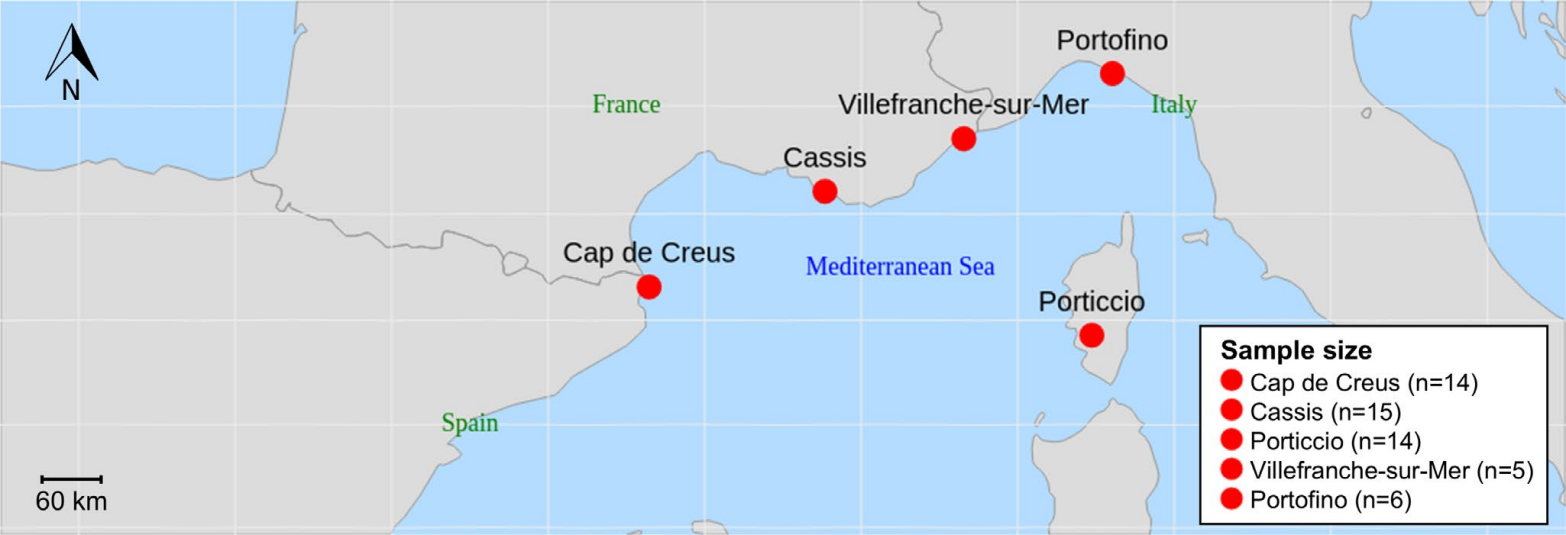
Geographic distribution of the sampling sites. Map of the Northwestern Mediterranean Sea showing the sampling locations: Cap de Creus, Spain (42.3°N, 3.3°E); Cassis, France (43.2°N, 5.5°E); Porticcio, Corsica, France (41.8°N, 8.8°E); Villefranche‐sur‐Mer, France (43.7°N, 7.3°E) and Portofino, Italy (44.3°N, 9.2°E). Samples were collected from visually healthy colonies at 30–40 m depth.

A total of 43 samples were collected from different colonies of *Corallium rubrum* in spring, between April and June 2013 at depths of 30–40 m from three sites: *Cap de Creus*, Spain (*n* = 14; 42°18′ N, 3°18′ E); *Cassis*, France (*n* = 15; 43°12′N, 5°28′E); and *Porticcio*, Corsica Island, France (*n* = 14; 41°50′N, 8°45′E) (Figure 1). At each site, visually healthy colonies were sampled, samples were rinsed with 0.2 μm‐filtered seawater and subsequently preserved in ice‐cold RNAlater RNA Stabilisation Solution (ThermoFisher Scientific) at 4°C. To broaden the geographic scope of the study and increase the sample size, data from Prioux, Ferrier‐Pagès, Del Campo, et al. (2025), which included *C. rubrum* samples collected in autumn (November) 2023 from *Villefranche‐sur‐Mer*, France (*n* = 5; 43.7°N, 7.3°E) and *Portofino*, Italy (*n* = 6; 44°18′N, 9°12′E) were incorporated (Figure 1). These samples were processed following the same protocol described in this study, increasing the total number of samples in the dataset to 54.

### 2.2 DNA Extraction and Amplification of the *18S rRNA* Gene

Approximately 1 cm^3^ of coral (comprising the soft tissue and the skeleton) was used for DNA extraction using the DNeasy PowerBiofilm Kit (Qiagen), with the following modifications: samples were placed into pre‐filled bead tubes containing 350 μL MLT buffer, 100 μL FB solution and 2 μL Proteinase K solution (600 U/mL; Qiagen), and bead‐beating was performed twice for 2 min at 30 Hz using a TissueLyser II (Qiagen). Two negative extraction control samples (i.e., extraction without sample material) were included in each batch of extractions to monitor contamination. The target amplicon size was approximately 600 bp, as described in previous studies (Prioux, Ferrier‐Pagès, Del Campo, et al. 2025; Prioux, Ferrier‐Pagès, Lamarca, et al. 2025). DNA concentrations were measured using an Invitrogen Qubit 4 Fluorometer (Fisher Scientific) and the Qubit dsDNA BR (Broad‐Range) Assay kit, and DNA samples were stored at −20°C.

The composition of the eukaryome was assessed through the amplification and sequencing of the V4 region of the *18S rRNA* gene using the primers 18S‐NonMet_Cr‐F (5′‐AAGTCTGGTGCCAGCASCC‐3′) and 18S‐NonMet_Cr‐R (5′‐TTTAAGTTTCAGCCTTGCGAT‐3′). These primers, adapted from Bower et al. (2004) and designed in Prioux, Ferrier‐Pagès, Del Campo, et al. (2025), minimise the metazoan *18S rRNA* gene amplification and have previously been validated for assessing the composition of the eukaryome associated with *C. rubrum* (Prioux, Ferrier‐Pagès, Del Campo, et al. 2025). Amplifications were performed in 25 μL reactions containing 5 μL of 5× Q5 buffer, 0.5 μL dNTPs, 1.25 μL of each primer (10 μM), 0.25 μL Q5 High‐Fidelity DNA Polymerase and 2 μL of template DNA (8 ng of DNA per sample). Cycling conditions were as follows: 98°C for 30 s; 35 cycles of 98°C for 10 s, 63°C for 30 s, 72°C for 30 s; and a final extension at 72°C for 2 min. Amplicon size was checked by agarose gel electrophoresis and purified with a GFX PCR and Gel Band Purification Kit (Illustra). DNA concentrations were measured using a Qubit fluorometer, and quality was assessed on an Agilent Bioanalyzer. Libraries, following Illumina's standard ‘16S Metagenomic Sequencing Library Preparation’ protocol (Illumina 2013), were diluted to 4 nM and sequenced at STAB‐VIDA (Portugal) on an Illumina MiSeq platform (2 × 300 bp). The fastq files containing the raw sequencing data have been deposited in the NCBI's Short Read Archive (SRA) under the BioProject accession number PRJNA1294578.

### 2.3 Bioinformatics Data Processing

Samples with fewer than 6000 reads (maximum read count observed in negative controls) were excluded from downstream analysis. After this filtering step, a total of 46 out of 54 samples remained for downstream processing. Amplicon data were processed in the R environment (version 4.3.2) using the DADA2 pipeline (v1.16; Callahan et al. 2016) and the Cutadapt tool (v3.1; Martin 2011). Raw paired‐end FASTQ files were filtered to remove reads containing ambiguous nucleotides (maxN = 0). Primer sequences were identified using exact matching and removed with Cutadapt prior to further processing. Reads were truncated according to quality profiles (truncLen = c(298,200), maxEE = c(2,2), rm.phix = TRUE). Filtered reads were dereplicated, and sequencing error rates were learned separately for forward and reverse reads. Denoising was performed using the DADA2 core algorithm with pool = ‘pseudo’ to increase sensitivity for rare variants. Due to the amplicon length (> 600 bp), paired‐end reads were concatenated instead of merged (mergePairs(…, justConcatenate = TRUE)) following data processing from Prioux, Ferrier‐Pagès, Del Campo, et al. (2025). Chimeric ASVs were detected and removed using the consensus method (removeBimeraDenovo()). Taxonomic assignment was generated using assignTaxonomy() and the Protist Ribosomal Reference (PR2) database (v5.0.0; Guillou et al. 2012). Reads assigned to Metazoa and Embryophyceae were filtered out (subset_taxa()). The number of reads per sample passing through the different steps of the pipeline, the ASV table, the taxonomic annotation and metadata tables are available in Tables S1–S4, respectively, and the sequences of the ASVs are available as Supporting Information 2. The percentage of ASVs assigned by taxonomic rank is Figure S2.

### 2.4 Analysis of the *18S rRNA* Gene Sequencing Data

Graphical and statistical analyses were conducted in the R environment, mainly using the R package *phyloseq* (McMurdie and Holmes 2013). Alpha diversity metrics, including observed number of ASVs (i.e., observed richness) and evenness (Shannon index, Simpson index and inverse Simpson index) were calculated using the R package *vegan* (Oksanen et al. 2012). Rarefaction curves for each sample were generated using the rarecurve() function (*vegan*) to assess sequencing depth saturation and confirm that all 46 samples analysed reached a plateau (Figure S1). Given the debate concerning the applicability of unrarefied versus rarefied data (McMurdie and Holmes 2014; Willis 2019), alpha diversity was also calculated on both rarefied and unrarefied ASV count tables (Table S5). As no substantial differences in patterns or results were observed between the two approaches, the analyses presented in the manuscript were performed on the unrarefied ASV count table to maximise statistical power and retain the full dataset (McMurdie and Holmes 2014). Differences in alpha diversity among sites were assessed using non‐parametric Kruskal–Wallis tests, followed by Wilcoxon rank‐sum tests for post hoc pairwise comparisons, with multiple‐testing correction applied. Pairwise Wilcoxon tests were performed with FDR correction (Table S5).

Compositional data analysis (CoDA) (Gloor et al. 2017; Quinn et al. 2018) was used to investigate the differences in the composition of the eukaryome (beta diversity and dispersion) between sites. Zero counts in the ASV table were replaced using a Bayesian‐multiplicative approach (cmultRepl() function from the R‐package *zCompositions* (Palarea‐Albaladejo and Martín‐Fernández 2015), and ASV abundances were subsequently subjected to centred log‐ratio (CLR) transformation (R‐package *microbiome*; Lahti et al. 2017)). An Aitchison distance matrix was then computed by calculating Euclidean distances between samples using the CLR‐transformed data. Hierarchical clustering was performed using the pvclust() function from the R‐package *pvclust* (Suzuki and Shimodaira 2006), using average linkage (UPGMA) and Euclidean distance. Statistical support for clustering was evaluated through multiscale bootstrap resampling with 1000 iterations. Differences in the eukaryome composition among sites and sampling years were further assessed using permutational multivariate analysis of variance (PERMANOVA), implemented with the adonis() function from the R‐package *vegan* (Oksanen et al. 2012). Because the factors ‘Site’ and ‘Year’ were confounding in our sampling design, we assessed their effect in separate models, thereby enabling a comparison of the proportion of variance each factor explains. Results including *p*‐values are reported in Table S6. Post hoc pairwise comparisons were conducted using the permanova_pairwise() function from the package *ecole* (Smith 2021), with *p*‐values adjusted for false discovery rate correction (Table S6). Differential abundance analyses were conducted to identify differentially abundant ASVs between sites, using the R‐package *ANCOM‐BC* (Lin and Peddada 2020) and the ancombc2() function with the parameters prv_cut = 0.1, alpha = 0.05, p_adj_method = ‘BH’ and pairwise = TRUE (version 02–2023; Table S7). Core members of the eukaryome (ASV and family level) across the entire dataset were identified using the core_members() function (arguments: detection = 0.001, prevalence = 0.95, include.lowest = TRUE) of the R‐package *microbiome* (Lahti et al. 2017; Table S8).

## 3 Results and Discussion

After quality filtering, 46 samples were retained for downstream analysis. This process reduced the number of replicates for some sites, resulting in the following distribution: *Cap de Creus* (*n* = 11), *Cassis* (*n* = 15), *Porticcio* (*n* = 12), *Villefranche‐sur‐Mer* (*n* = 4) and *Portofino* (*n* = 4). Nevertheless, the remaining dataset comprised 2404 ASVs and a total of 3,417,757 reads, with per‐sample read counts ranging from 46,868 to 183,202, providing a comprehensive overview of eukaryome diversity across the five study sites (Tables S1 and S2). Rarefaction analyses confirmed sequencing depth saturation for all samples, with curves reaching a plateau (Figure S1), indicating that sequencing effort was sufficient to capture the majority of ASV diversity.

**FIGURE 2:**
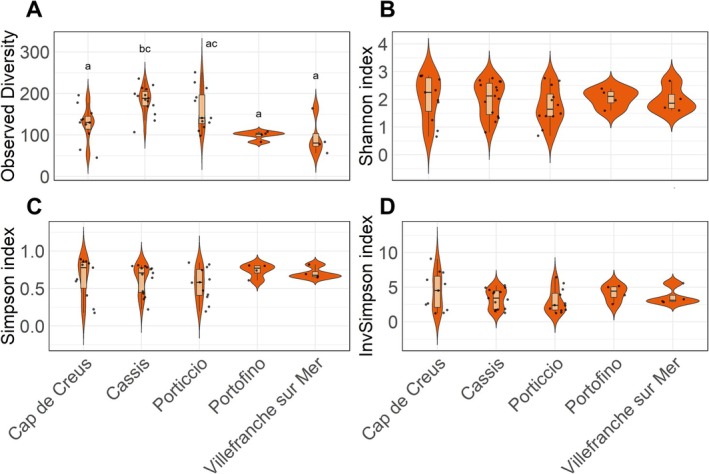
Alpha diversity metrics across sampling sites. Observed richness (ASV level) (A), Shannon index (B), Simpson index (C) and Inverse Simpson index (D). Graphs are showing value distributions, their median and interquartile range per sampling site. Groups assigned with the same letters are not statistically different.

Observed number of ASVs varied significantly between sites (Kruskal–Wallis test; Statistic = 18.72, DF = 4, *p* = 0.00089; Table S5 and Figure 2A). Pairwise comparisons using Wilcoxon rank‐sum tests with FDR correction showed that *Cassis* had the highest observed number of ASVs (mean = 183.7 ASVs; Table S5 and Figure 2A), which was significantly different from all other sites except *Porticcio*. The high level of diversity observed in *Cassis* is likely attributable to local factors, with geographical and/or temporal variables potentially driving this increased richness. Alpha diversity metrics calculated on rarefied datasets yielded comparable results (Table S5), supporting the robustness of these patterns and justifying the use of unrarefied data to maximise statistical power and retain the full dataset (McMurdie and Holmes 2014). In contrast, evenness, as measured by the Shannon index, did not differ significantly between sites (Table S5 and Figure 2B), suggesting that all coral eukaryomes are composed of a few dominant microeukaryotes alongside many low‐abundance taxa. Similarly, no significant differences were observed for the Simpson and inverse Simpson indices (Table S5 and Figure 2C,D), indicating that the overall community structure was relatively consistent despite variations in species richness. Altogether, these analyses show that the diversity of the *C. rubrum* eukaryome varies between sites, with site‐specific differences primarily driven by observed richness.

**FIGURE 3:**
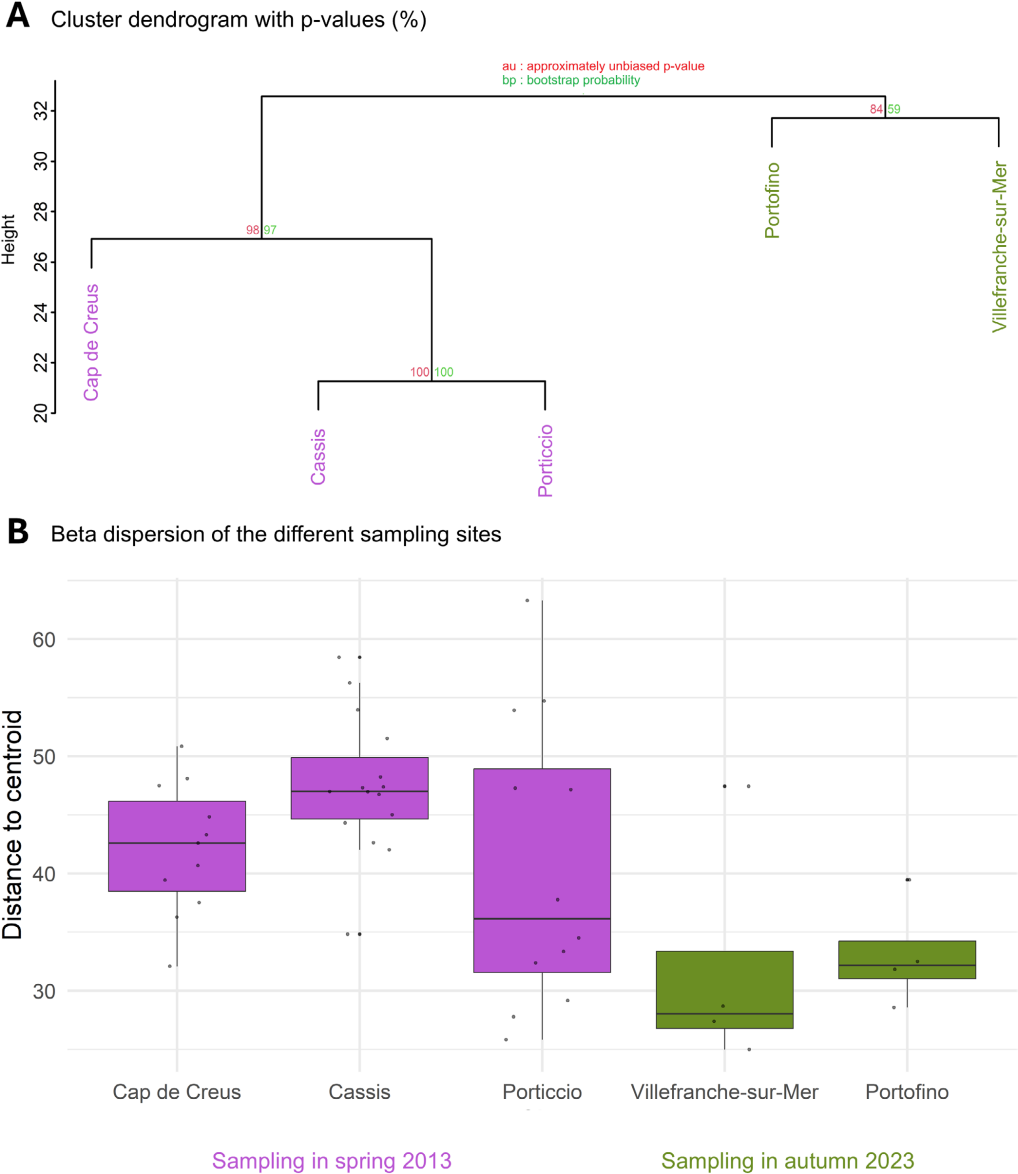
Structure of the red coral eukaryome depending on the sampling site and year. Hierarchical cluster analysis on the Aitchison distance matrix based on the eukaryome structure (CLR‐transformed ASV data) (A) and beta dispersion (within‐site variability) of the samples based on the community composition at the ASV level. Boxplots display value medians and interquartile ranges for each site (B).

The hierarchical cluster analysis based on the eukaryome composition revealed two clusters of sites, with western sites sampled in spring 2013 (*Cap de Creus*, *Cassis*, *Porticcio*) clustering separately from eastern sites sampled in autumn 2023 (*Villefranche‐sur‐Mer*, *Portofino*) (Figure 3A). Moreover, the eukaryome structure was significantly different between sites (PERMANOVA; *R* ^2^ = 0.14, *F* = 1.7, *p* = 1 × 10^−3^; Table S6 and Figure 3A) and between sampling years (PERMANOVA; *R* ^2^ = 0.04, *F* = 1.9, *p* = 1 × 10^−3^; Table S6 and Figure 3A). Comparison of the resulting *R* ^2^ values revealed that the model with ‘Site’ as a factor explains 14% of the total variance in community composition, while ‘Year’ accounts for 4%. This result suggests that variation in the structure of the microeukaryotic community between the samples is mainly explained by geography. Nevertheless, the percentage of variance explained remains modest, indicating that a substantial portion of community variation is likely attributable to unmeasured environmental factors. Furthermore, intra‐site variability was significantly higher in samples from spring 2013 than autumn 2023 (Betadispersion; ANOVA; *p* = 0.005; Table S6 and Figure S2) and more precisely higher in *Cassis* than in *Villefranche‐sur‐Mer* and *Portofino* (Tukey test; *p* = 0.017 and *p* = 0.030; Table S6 and Figure 3B). Although physicochemical parameters were not measured, regional environmental differences, together with the temporal factor, likely underlie the observed structuring of red coral eukaryome. Previous studies indicate that hydrodynamics, nutrient availability, and local thermal regimes can modulate coral‐associated microbiota (Sweet and Séré 2016; Martinez 1980; Mandal 2024; Maire et al. 2022), which could potentially explain these patterns.

**FIGURE 4:**
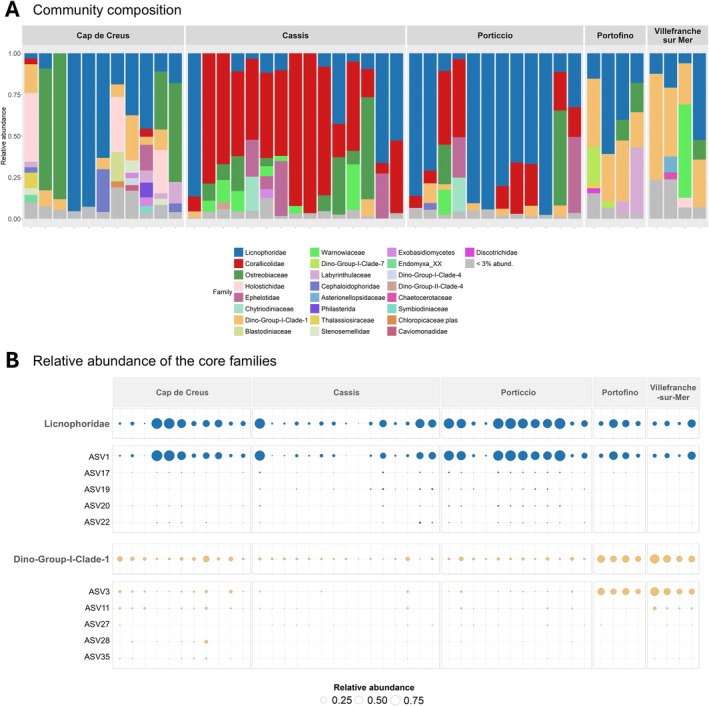
Composition of the eukaryome of *C. rubrum* across the sampling sites. Composition of the whole microeukaryotic community of the red coral across the samples (Family level). In each sample, families with a relative abundance lower than 3% were grouped (A). Relative abundance of core families and their top five ASVs across all samples (B).

Analysis of the core eukaryome revealed the consistent presence of one ASV, ASV1‐Licnophoridae, representing a substantial proportion of the eukaryome at all sites (mean relative abundances per site: 59% in *Porticcio*, 51% in *Cap de Creus*, 36% in *Portofino*, 24% in *Villefranche‐sur‐Mer* and 17% in *Cassis*; Table S7 and Figure 4A,B). Despite high fluctuations in abundance, the constant association of these ciliates with *C. rubrum* suggests an important ecological role within the red coral holobiont. Licnophoridae have been previously reported in other corals, including the Mediterranean octocoral *Paramuricea clavata* (Bonacolta et al. 2024) and the tropical hexacoral *Pocillopora damicornis* (Clerissi et al. 2018), although their functional role remains uncharacterised. Species of Licnophoridae are known predators of other ciliates (Sweet and Séré 2016), potentially contributing to their ecological role within coral holobionts. Members of the Dino‐Group I Clade 1 family were also detected in all red coral samples, generally at lower abundances, with relatively higher levels in *Portofino* and *Villefranche‐sur‐Mer* (Table S7; Figure 4A,B). This family has also been identified in other corals, including *P. damicornis* and *P. clavata* (Clerissi et al. 2018; Bonacolta et al. 2024). The latter study showed that heat‐resistant gorgonians harboured eukaryomes enriched with Dino‐Group I Clade 1, suggesting a potential role in stress tolerance, although this remains to be investigated.

**FIGURE 5:**
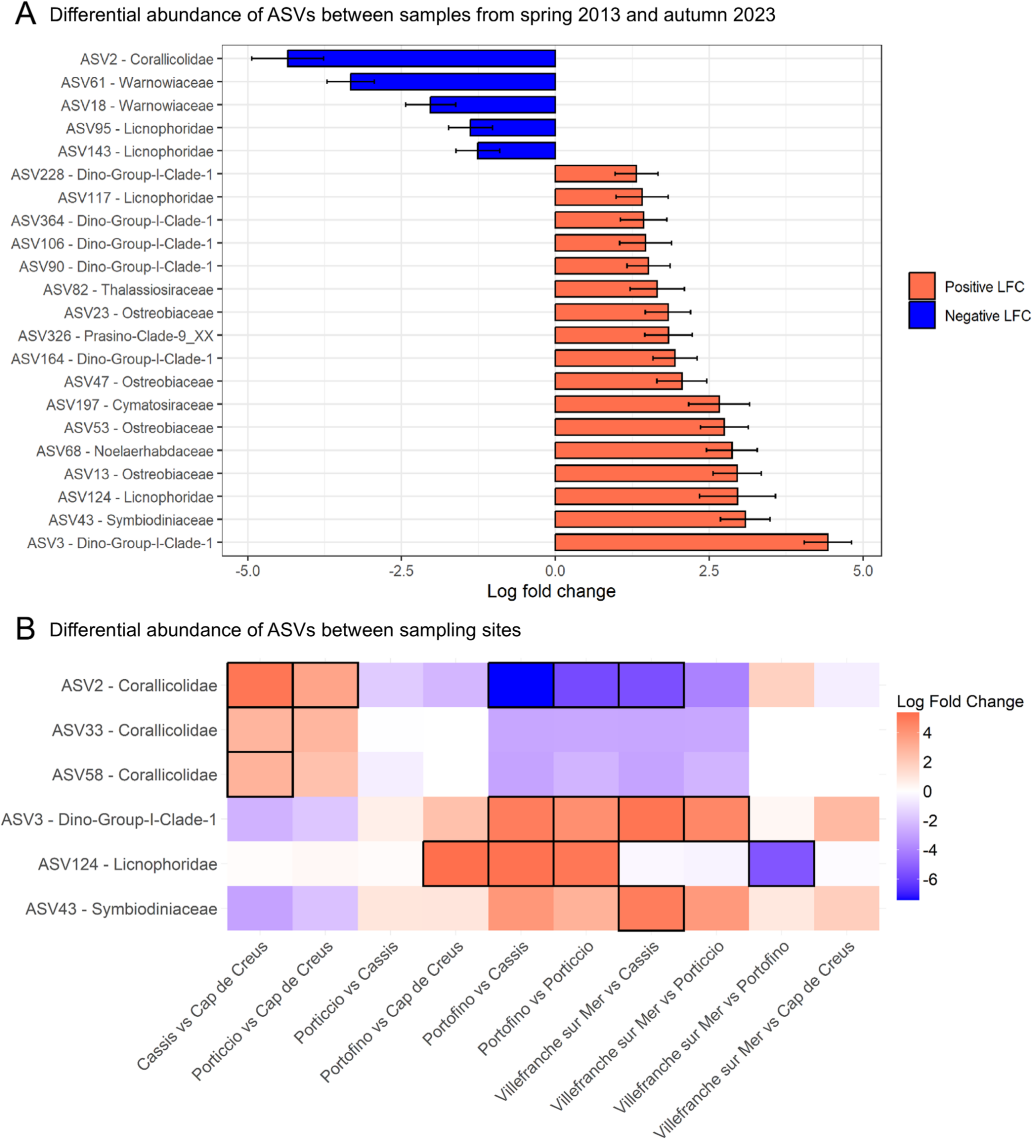
ASVs differentially abundant (ANCOM‐BC results) between the sampling years (A) and sites (B). Differences in relative abundance are calculated as a log_2_ fold change (base 2). In (A), positive values indicate taxa significantly more abundant in samples from autumn 2023 than spring 2013 and negative values taxa less abundant in these samples. In (B), statistically significant values are framed in black.

Dino‐Group I Clade 1, though ubiquitous, was significantly more abundant in samples from autumn 2023 (*Villefranche‐sur‐Mer* and *Portofino*), especially ASV3‐Dino Group‐I Clade‐1 (mean of 37% and 33%, respectively; ANCOM‐BC; Tables S8 and S9; Figures 4A and 5A,B). These relative differences may contribute to the observed clustering patterns, which are likely shaped by environmental fluctuations associated with site localization (western vs. eastern) and/or temporal factors (seasonal variation between spring and autumn, as well as differences between 2013 and 2023).

Additionally, some taxa were differentially abundant depending on the sampling site, contributing to the site‐specific structure of the red coral eucaryome. For example, samples collected in *Cap de Creus* showed high relative abundances of ASV5‐Ostreobiaceae (mean relative abundance of 15%; ANCOM‐BC; Figure 4A and Figure 5), a taxon that was already recovered in similar abundance in laboratory‐maintained red coral colonies (Prioux, Ferrier‐Pagès, Lamarca, et al. 2025). In contrast, samples collected in *Cassis* and *Porticcio* showed high relative abundances of ASV2‐Corallicolidae (ANCOM‐BC; Table S7; Figure 3), which accounted for a mean of 55% and 18% of the community, respectively (Figures 4A and 5). Apicomplexan alveolates from the family Corallicolidae are widely distributed and were found in high prevalence across all major groups of corals (Kwong et al. 2019, 2021; Bonacolta et al. 2023, 2024). From a total of 43 samples representing 38 coral species, a large genomic survey recovered the apicomplexan *Type‐N 18S rRNA* and *ARL‐V 16S rRNA* genes in 62% and 83% of the coral samples, respectively (Kwong et al. 2019), highlighting its potential important role in coral holobionts. The sequence of ASV2 was identical (100% sequence identity) to the sequence of a Corallicolidae ASV associated with heat‐stressed *C. rubrum* colonies (Prioux, Ferrier‐Pagès, Del Campo, et al. 2025), and closely related to ASV sequences found in *P. clavata* colonies (99.21% sequence identity; Bonacolta et al. 2024). This latter study hypothesised that this taxon may exhibit context‐dependent functional roles within coral holobionts, potentially transitioning from a symbiotic or commensal lifestyle to an opportunistic/parasitic one in response to the host physiological state or environmental stressors.

Overall, our results showed that the composition of the eukaryome varied significantly depending on environmental parameters, including geographical and temporal parameters. This contrasts with the bacterial community associated with the red coral, which has been shown to be relatively stable across geographical and temporal scales (van de Water et al. 2016). Furthermore, our findings indicate that the eukaryome composition was relatively variable between the samples of the same sites. This high inter and intra‐site variability suggests that local environmental conditions likely play a significant role in shaping the composition of the red coral eukaryome. The relative abundance of certain microeukaryotes might fluctuate in response to environmental factors such as temperature (Sweet and Séré 2016; Martinez 1980; Mandal 2024; Maire et al. 2022). Prioux, Ferrier‐Pagès, Lamarca, et al. (2025) also showed that the eukaryome of laboratory‐maintained red coral colonies responded more rapidly to thermal stress than the bacterial microbiome. This heightened sensitivity suggests either a greater responsiveness to environmental fluctuations with a potentially greater flexibility to adapt to local conditions or a more tenuous, less obligatory relationship between microeukaryotes and the coral host, in contrast to the more stable bacterial symbionts. In this study, the absence of environmental context prevented us from explaining this variability which could be attributed to factors such as temperature, site‐specific resource availability, or interactions with surrounding microbial communities (Brannock et al. 2016; Pernice et al. 2013; Zhang et al. 2018). Future research exploring changes in the eukaryome composition over different seasons and under controlled environmental conditions will be critical to better understand how the eukaryome associated with *C. rubrum* is structured.

## 4 Conclusion

Our findings indicate that the composition of the eukaryome of the red coral *C. rubrum* is highly influenced by environmental conditions. Variations in species richness, community structure, and dominant microeukaryotes across geography and time highlight the high sensitivity of the eukaryome to the environmental context. While site‐specific microeukaryotes may not be essential to the coral's core functioning, they could offer context‐dependent benefits, such as assimilation of local resources or protection against local stressors. In contrast, certain families, such as Licnophoridae and Dino‐Group I Clade 1, were consistently detected across all sites, suggesting that they may represent key symbiotic partners within the red coral holobiont. Future research should aim to elucidate the functional roles of these potentially critical taxa and investigate their responses to environmental variation, to enhance our understanding of holobiont stability and coral health in the face of global change. These results will be critical not only for understanding red coral resilience, but also for informing conservation strategies under accelerating climate stressors.

## Author Contributions

The study was designed by R.T.‐P., C.F.‐P., C.P. and D.A. D.F. and C.P. performed laboratory experiments and analysed the data. D.F., C.P., R.T.‐P. and C.F.‐P. wrote the manuscript with feedback and input from all co‐authors.

## Conflicts of Interest

The authors declare no conflicts of interest.

## Supporting information


**Figure S1:** Rarefaction curves for each sample.
**Figure S2:** Beta dispersion (within‐site variability) of the samples based on the community composition at the ASV level. Boxplots display value medians and interquartile ranges for each sampling year.

**Data S1:** Supporting Information 1.

**Data S2:** Supporting Information 2.

## Data Availability

The data that support the findings of this study are openly available in NCBI's Short Read Archive (SRA) at https://www.ncbi.nlm.nih.gov/bioproject/PRJNA1294578, reference number PRJNA1294578.
