# Supplementary material for: Unveiling the Microeukaryotic Landscape of the Red Coral Corallium rubrum Across the Northwestern Mediterranean Sea
Source: Environ Microbiol Rep. 2025 Nov 25;17(6):e70227. doi: 10.1111/1758-2229.70227 (PMC12645308; doi:10.1111/1758-2229.70227)
Supplement: Supplementary file 1 — Figure S1: Rarefaction curves for each sample. Figure S2: Beta dispersion (within‐site variability) of the samples based on the community composition at the ASV level. Boxplots display value medians and interquartile ranges for each sampling year. [file EMI4-17-e70227-s002.docx]

**Figure S1: Rarefaction curves for each sample.**


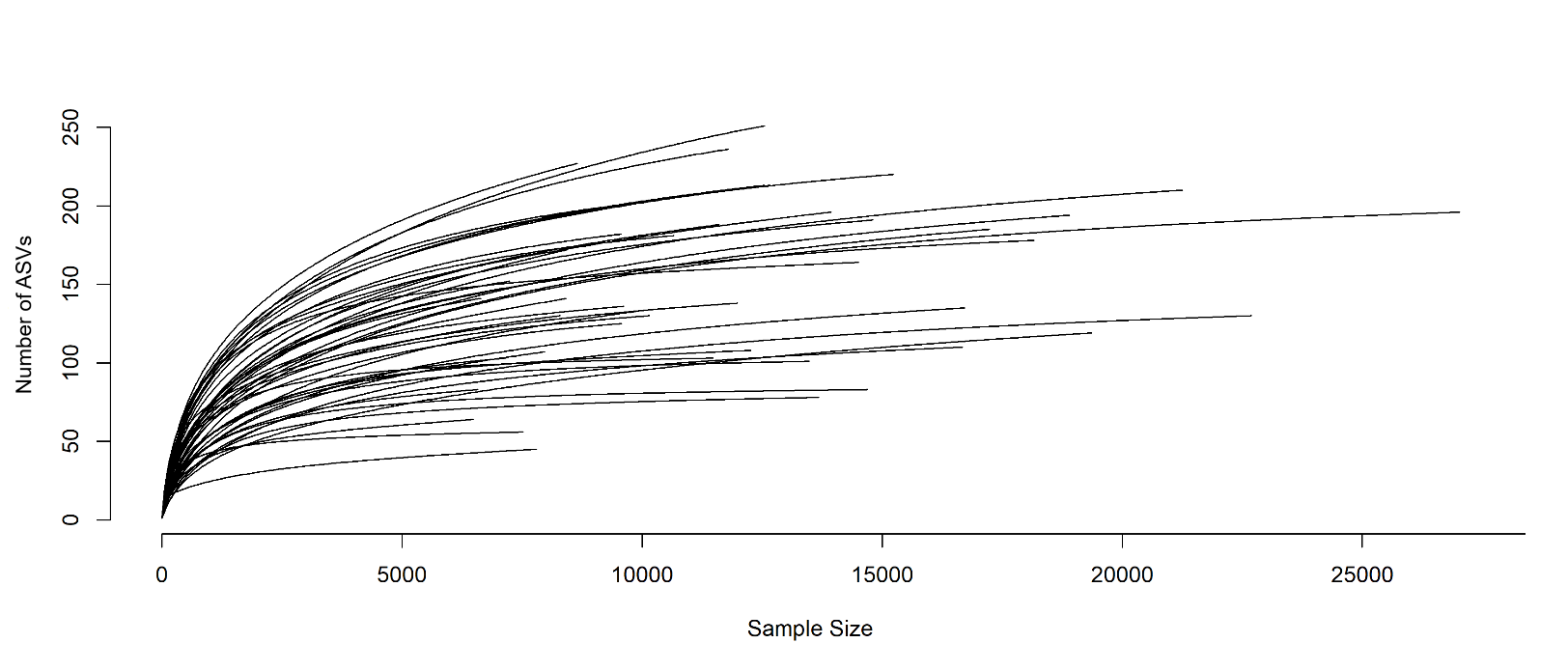

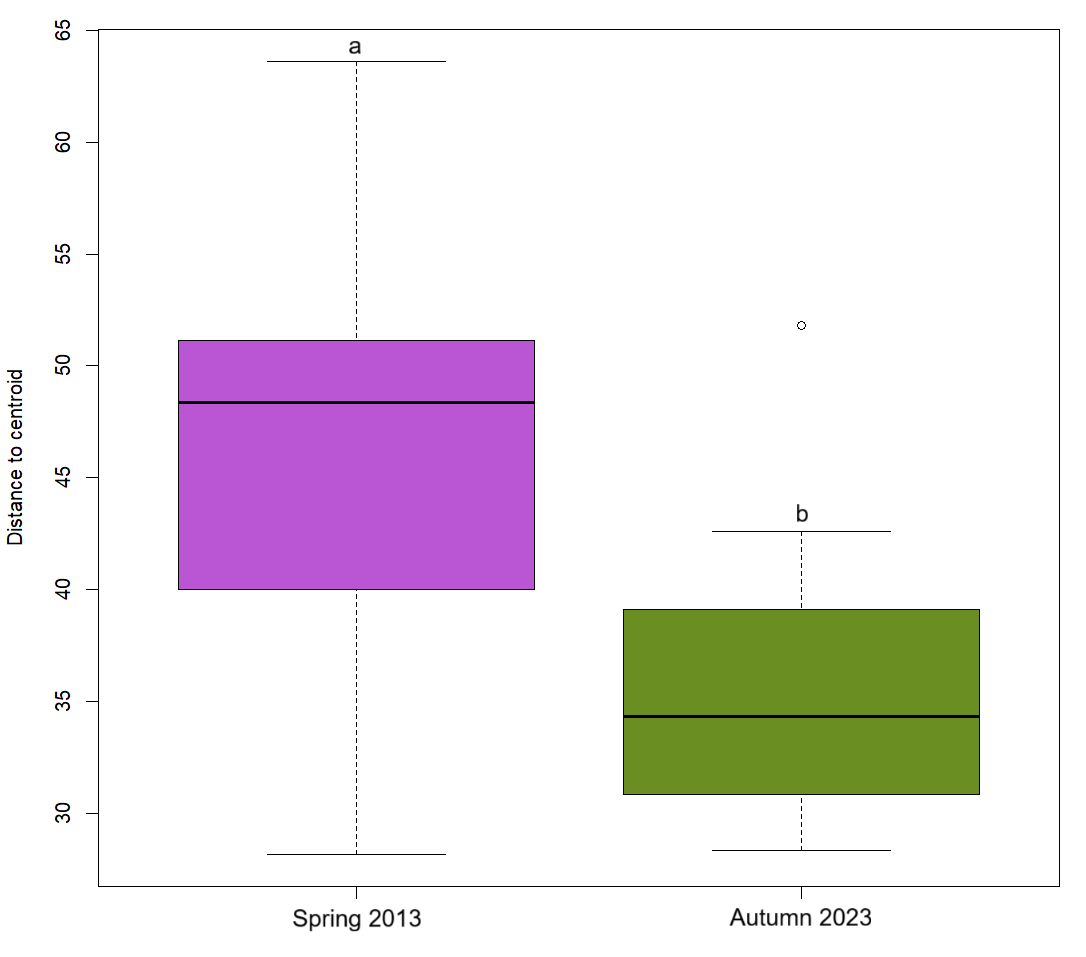


**Figure S2: Beta dispersion (within-site variability) of the samples based on the community composition at the ASV level. Boxplots display value medians and interquartile ranges for each sampling year.**
